# Preliminary results of intracranial aneurysm treatment with derivo2heal embolization device

**DOI:** 10.1007/s00234-024-03387-y

**Published:** 2024-07-01

**Authors:** J. Rueckel, Y. Ozpeynirci, C. Trumm, C. Brem, M. Pflaeging, T.D. Fischer, T. Liebig

**Affiliations:** Institute of Neuroradiology, University Hospital, LMU Munich, Munich, Germany

**Keywords:** Intracranial aneurysm, Flow diversion, Derivo 2heal

## Abstract

**Introduction:**

The Derivo 2 Heal Embolization Device (D2HED) is a novel flow diverter (FD) providing a fibrin-/heparin-based surface coating aiming at lower thrombogenicity. We evaluate periprocedural aspects and preliminary aneurysm occlusion efficacy for intracranial aneurysm treatment.

**Methods:**

Thirty-four D2HEDs deployments (34 aneurysms, 32 patients) between 04/2021 and 10/2023 were analyzed. All patients were under dual antiplatelet therapy (dAPT). Periprocedural details, adverse events, and follow-up (FU) imaging were reviewed by consultant-level neuroradiologists. Complication rates and aneurysm occlusion efficacy are compared with performance data of other FDs based on literature research.

**Results:**

Each intervention succeeded in the deployment of one D2HED. Significant and/or increased intraaneurysmal contrast stagnation immediately after D2HED deployment was seen in 73.5% of cases according to O’Kelly-Marotta (OKM) grading scale. Clinically relevant early adverse events occurred in three patients: Among them two cases with fusiform aneurysms in the posterior circulation (ischemic events, early in-stent-thrombosis) and one patient (ischemic event) out of the majority of 31 treated internal carotid artery aneurysms (3,2%). Regarding mid-term FU (> 165 days), one aneurysm did not show progressive occlusion presumably caused by a prominent A1 segment arising from the terminal ICA aneurysm itself. Apart from that, mid-term complete / partial occlusion rates of 80% / 20% could be demonstrated.

**Conclusion:**

Our case series - although suffering from restricted sample size - suggests a potential effectiveness of D2HED in managing intracranial aneurysms. Further studies with larger samples are warranted to quantify long-term occlusion efficacy and the impact of antithrombogenic surface coating on the necessary (d)APT.

## Introduction

Flow diverter stents (*flow diverters, FDs*) have been established as an effective way to treat intracranial aneurysm with continuously expanding indications for use [1, 2]. In contrast to intrasaccular endovascular technologies, FDs are designed for parent vessel reconstruction, flow disruption and redirection away from the aneurysmal lumen as well as to promote endothelial growth following intraaneurysmal thrombosis and/or aneurysm regression [3] – even if the exact mechanism of aneurysm healing is not completely understood [4]. The possibility of parent vessel reconstruction especially simplified the treatment of large/giant, broad-neck and fusiform aneurysms [1, 5, 6]. Nevertheless, a relevant disadvantage of stent-assisted vessel reconstruction remains the requirement of long-term antiplatelet therapy aiming to prevent stent-thrombosis, side-branch occlusion and distal emboli [7].

Following the first FD approved by the Food and Drug Administration in 2011 (*Pipeline Embolization Device, Covidien, Mansfield, Massachusetts, USA*), a variety of different FDs have been introduced over the last few years. These share mutual differences from conventional stents, such as higher metal coverage and flexibility for improved parent vessel wall apposition along with low radial forces during expansion but high chronic outward radial strength once fully opened [4, 8]. A reduction in thrombogenicity by surface modification is a concept pursued by various manufactures in the latest FD generation. Manufacturer-specific surface modifications are, e.g., the *shield technology* (covalently bonded synthetic phosphorylcholine to implant surface, e.g. *Pipeline Flex / Vantage Embolization Devices, Medtronic, Dublin, Ireland*), a poly(2-methoxyethylacrylate) coating (*FRED X, Microvention, Aliso Viejo, US*), hydrophilic coating *(Phenox HPC, p48/p64, Phenox, Bochum Germany)*, and *heal technology* (covalently bonded fibrin/heparin to implant surface, e.g. *Derivo2Heal Embolization Device, Acandis, Pforzheim, Germany*) [9–12].

The objective of this monocenter study is to share one of the first available clinical experiences with the *Derivo2Heal Embolization Device* (D2HED [13]), for the treatment of intracranial aneurysms. There is only one similar case series published so far, nevertheless with a significantly lower number of only 9 patients included and especially only 2 patients with a follow-up period of at least six months [14]. Building upon that, we hereby provide the currently largest case series and the only one focussing also on mid-term follow-up results.

The D2HED is a woven, self-expanding flow diverter that is made of nitinol composite wires with a platinum core (DFT, drawn filled tube) and provides a heparin-/crosslinked fibrin-based nano-coating (*heal technology)*. The available FD sizing is approved for parent vessel diameters from 1.5 up to 8 millimeters [13]. We aimed to evaluate device efficacy regarding periprocedural feasibility, early and late complications, thereby also by analyzing mid-term follow-up imaging.

## Methods

Approval of the institutional ethics committee was obtained for this study (reference number 23–0735). Patients’ informed consent for the retrospective intervention data evaluation was waived.

### Data collection

Thirty-four D2HED deployments between 04/2021 and 10/2023 were included, thereby addressing 34 intracranial aneurysms in 32 patients. Patients have been retrospectively identified by data research in our prospectively kept database. We aimed for consecutive D2HED patient inclusion, with the only retrospective inclusion criterion being the selected D2HED flow diverter (various other flow diverters were used for other patients in the same period) and no additional exclusion criteria applied (all D2HED treatments are included in the analysis). There were no general criteria that systematically favored the use of D2HED over other flow diverters - the flow diverter selection has been made based on neuroradiologists’ preferences and available stock sizes.

Patient demographic data and aneurysm characteristics based on digital subtraction angiography (DSA) with 3D-rotational angiography have been evaluated. Aneurysm size was quantified regarding dome height and neck width, adjacent parent vessel diameters were quantified at the FD landing zones. Periprocedural data was collected from interventional reports as well as corresponding image documentation, pre- and postinterventional aneurysmal contrast stasis was quantified by O’Kelly-Marotta (OKM) grading scale [15, 16]. Peri-/postinterventional complications have been recorded (also based on postinterventional imaging, which during the ongoing hospitalization, however, was only reserved for cases of suspected complications and was not routinely performed). Regular follow up imaging – if available – was reviewed for patency of parental and covered side-vessels, aneurysm occlusion, intimal hyperplasia and complications such as ischemic events.

Clinical information was extracted from the medical records as documented by the physicians during the corresponding hospital stays and, in case of outpatient-based non-invasive follow-up CTA/MRA, during the follow-up consultations. Periinterventional angiography and follow-up imaging including the corresponding reports have been reviewed by consultant-level neuroradiologists for structured re-evaluation and data preparation with the primary oversight from a consultant who was involved in the interventions only to a very minor extent (treating physician in two of the 34 included interventions).

### Interventional procedures

Indications for aneurysm treatment were based on an interdisciplinary consensus involving neurosurgeons and neuroradiologists. Interventional procedures have been performed via transfemoral access in general anesthesia and under full heparinization according to our institutional standards. The standard approach involved a 6F femoral sheath, a 6F guiding catheter with an additional 5F-SOFIA intermediate catheter in 8 cases and FD delivery through a Phenom 0.027” microcatheter placed at the distal end of the landing zone that has been withdrawn during deployment. Microcatheter manipulation was used in 4 cases and balloon-remodeling in 6 cases in order improve vessel wall apposition after FD deployment. Parental vessel wall apposition was assessed by DSA, frequently with additional flat panel CTA including thin slice reconstruction. Five interventionalists (two senior consultants, three junior consultants) participated in the interventions. Early procedure-related complications were recorded based on periinterventional documentation and/or postinterventional imaging during ongoing hospitalization.

### Antiplatelet therapy (APT)

The antiplatelet protocol as described below for the D2HED patients of this study did not differ from our institutional routine protocol used for both coated and non-coated flow diverters in the same way.

- Patients with incidental aneurysms have been prepared with two antiplatelet agents (ASA [100 mg/d] + Clopidogrel [75 mg/d] or Ticagrelor [2 × 90 mg/d] or Prasugrel [10 mg/d]) starting five days before elective intervention with the individual antiplatelet combination based on the interventionalist’s choice and drug response testing. Interventions were started after sufficient antiplatelet drug response was proven by a *Multiplate Analyzer (Roche, Basel, Switzerland).* Maintenance dual APT (ASA + ADP receptor antagonist) was continued for three to six months, followed by permanent single APT by ASA.
- One patient with a ruptured aneurysm received periprocedural APT by tirofiban infusion, followed by maintenance dual APT as described above for incidental aneurysm (with loading doses of ASA & ADP receptor antagonist four hours before tirofiban infusion was stopped).
- Two patients with large fusiform aneurysms received the dual APT maintenance protocol supplemented by an anticoagulant (Heparin followed by Dabigatran/Apixaban until imaging follow-up).

### Follow-up & aneurysm occlusion evaluation

Regular follow-up imaging was performed by computed tomography angiography (CTA), magnetic resonance imaging (MRI) or digital subtraction angiography (DSA). The choice of imaging follow-up recommendations was individually made by the treating neuroradiologists in consultation with the patients. Patency of parent vessel as well as stent covered side branches, presence of intimal hyperplasia, aneurysm occlusion and evidence of ischemic/thromboembolic events were evaluated based on available imaging. In case of DSA follow-up, remaining aneurysm perfusion was quantified based OKM grading (and in comparison with the periinterventional final DSA control runs). In case of MRA follow-up, remaining aneurysm perfusion was qualitatively assessed.

## Results

### Patient demographics & aneurysm characteristics

A detailed overview of patient and aneurysm characteristics is illustrated in Table 1. Thirty-four aneurysms, among them one ruptured, in 32 patients (average age 58+/-14 years with a range of 25-84yo, 72% female sex) were included. Two aneurysms have been previously treated (1x retreatment of a neck remnant after coiling, 1x retreatment of an aneurysm recurrence after clipping). In all interventions, only one D2HED flow diverter was finally deployed without simultaneous implantation of other endovascular occlusion material. The internal carotid artery (ICA) was the most common aneurysm location (*n* = 31, 91%); another three aneurysms were in the posterior circulation (1x V4 segment, 2x basilar artery). A majority of 28 aneurysms was saccular with a mean dome height of 5.7+/-3.2 mm (range 2.7–14.1 mm) and a mean neck width of 4.3+/-1.5 mm (range 1.4–7.0 mm), six aneurysms were classified as fusiform or fusiform-dysplastic with diameters of 13.8+/-5.4 mm (range 9.3–23.0 mm), 13 aneurysms (38.2%) were of irregular or lobular morphology. In seven cases, there were angiographically visible side branches arising from the aneurysm sac.

**Table 1:** Aneurysm characteristics

Aneurysm #	Comment*	Localization	Side Vessel From Aneurysm	Dome Height [mm]	Neck Width [mm]	Irregular Lobulated
1	recurrence after clipping	ICA paraophthalmic (r)	-	5.9	4.3	yes
2		ICA paraophthalmic (I)	-	2.7	3.8	no
3	-	ICA paraophthalmic (I)	-	5.4	5.8	yes
4	aneurysm remnant after coiling	ICA paraophthalmic (I)	-	13.4	5.5	no
5	-	ICA paraophthalmic (I)	-	2.9	2.4	no
6	-	ICA paracavernosal (I)	-	6.0	6.3	yes
7	-	BA (sidewall)	-	7.6	6.5	yes
8	-	ICA supraophthalmic (I)	A1 adjacent to aneurysm neck	3.3	4.9	no
9	-	ICA paraophthalmic (r)	-	5.6	4.0	yes
10	ruptured	ICA paraophthalmic (r)	-	3.5	3.6	no
11	-	ICA supraophthalmic (r)	AchA	fusiform max. ca. 11,6 mm diameter		no
12	-	ICA paracavernosal (r)	-	6.6	7.0	no
13	-	ICA paraophthalmic (I)	-	2.8	3.0	yes
14	-	ICA infraclinoidal (I)	-	3.9	2.5	no
15	-	ICA paraophthalmic (r)	-	3.1	1.4	yes
16	-	BA (distal)	SCA / PCA (bilateral)	fusiform max. ca. 11,5 mm diameter		no
17	-	ICA supraophthalmic (I)	-	2.9	4.0	no
18	-	ICA paracavernosal (I)	-	fusiform-dysplastic max. ca. 9,6 mm diameter		yes
19	.	ICA paracavernosal (r)	-	12.9	6.3	yes
20	-	ICA supraophthalmic (r)	AchA	fusiform-dysplastic max. ca. 9,3 mm diameter		yes
21	-	ICA supraophthalmic (r)	AchA	5.2	3.3	no
22	-	ICA paraophthalmic (I)	-	3.9	6.8	yes
23	-	ICA paraophthalmic (r)	-	8.2	3.2	yes
24	-	V4 segment (I)	PICA	fusiform max. ca. 17,7 mm diameter		no
25	-	ICA paraophthalmic (I)	-	5.8	4	no
26	-	ICA paraophthalmic (I)	-	4.9	4	yes
27		ICA supraophthalmic (r)	-	6.4	3.4	no
27	retreatment / FD extension	ICA supraophthalmic (r)	-	6.4	3.4	no
28	-	ICA paraophthalmic (I)	OA from aneurysm neck	4.9	3.4	no
29	-	ICA paracavernosal (I)		fusiform-dysplastic max. ca. 23 mm diameter		no
30	-	ICA paraophthalmic (r)	OA from aneurysm neck	3.8	3.1	no
31	-	ICA supraophthalmic (r)		2.5	3.6	no
32	-	ICA supraophthalmic (I)		3.8	2.7	no
33	-	ICA paracavernosal (r)	OA from aneurysm neck	14.1	7.0	no
34		ICA paraophthalmica (r)		2.7	1.7	no

Thirty-four aneurysms of 32 patients have been treated by 34 FD-deploying interventions: Aneurysm #27 was treated in the early postinterventional course by a second FD based on progressive proximal fishmouthing of the initially deployed one. Two adjacent ICA aneurysms #33/34 of the same patient (paracavernosal & paraophthalmic) were treated with one FD. The bilateral ICA aneurysms #1/2 belong to the same patient and have been treated in separate procedures. *First time therapy of an unruptured aneurysm unless commented otherwise. FD, flow diverter; ICA, internal carotid artery; BA, basilar artery; AchA, anterior choroidal artery; SCA, superior cerebellar artery; PICA, posterior inferior cerebellar artery; PCA, posterior cerebral artery; OA, ophthalmic artery; left; r, right

### Periprocedural treatment evaluation

**Fig. 1:**
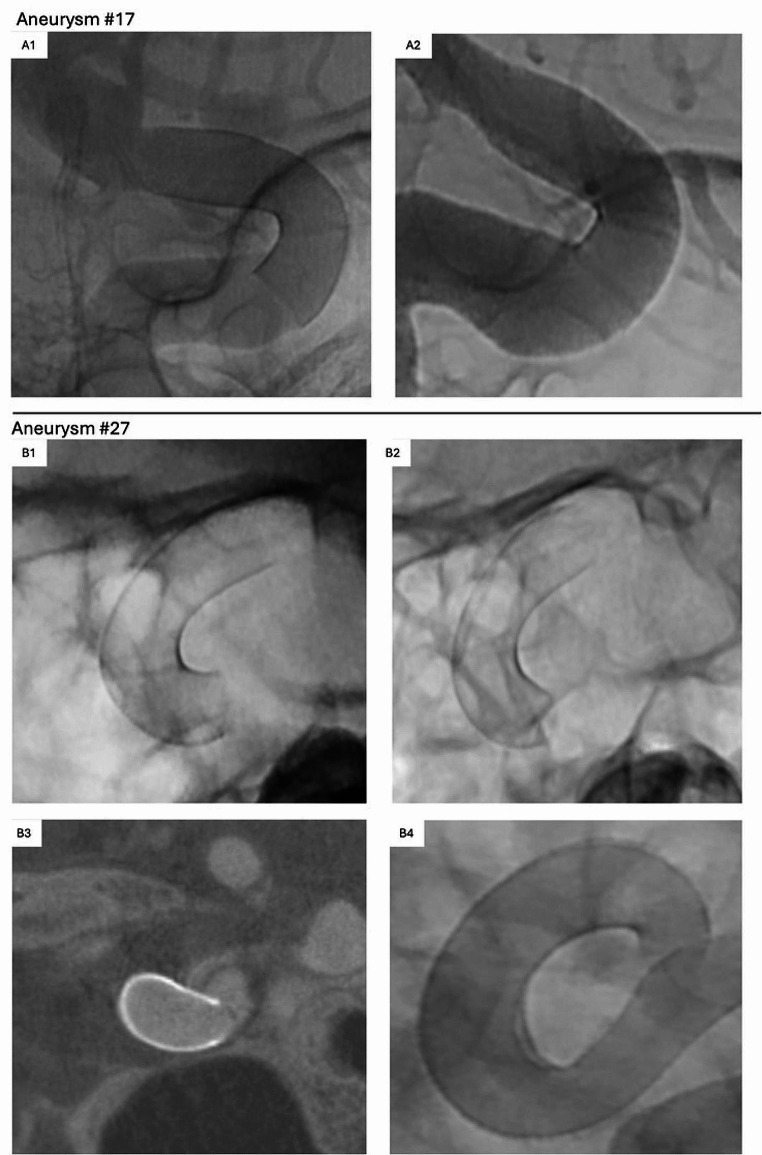
Device Visibility & Fish Mouthing Example. *(A1/2)*: Postinterventional control after FD deployment with incomplete apposition of the proximal FD to the parental vessel curvature (A1) with FD retraction and complete vessel wall adaptation based on follow-up DSA 257 days after intervention (A2). *(B1-4)* Progressive proximal fish mouthing: Initial post-deployment projection (B1), compared with a control projection the following day (B2) after initial suspicion was noticed by a CT at the first postinterventional day; followed by the decision for a second FD deployment with FPCTA before the second FD deployment (B3) and control angiogram after second FD deployment (B4). FD, flow diverter; DSA, digital subtraction angiography

Periprocedural characteristics and measurements are elucidated in Table 2 (34 interventions, 34 aneurysms in 32 patients). Satisfactory visibility of the D2HED under fluoroscopy is illustrated in Fig. 1. Final device deployment with complete aneurysm coverage was successful in all cases, in four however (4/40 FD deployment attempts, 10%) the initial deployment attempt failed for technical reasons (three FDs did not show a satisfactory opening behavior, one FD retrieval failed before intended re-positioning, all technical failures were connected to relatively long stent sizes of 25–60 mm). Mechanical manipulation by microwire / microcatheter (*n* = 4) and/or balloon angioplasty (*n* = 6) was performed for better wall apposition in ten cases (29%). An incomplete wall apposition of the FD landing zones wall was only observed for the proximal end and persisted in 11/34 procedures (32%) - predominantly associated with tight vessel curvature in 10/11 cases. All unavoidably covered side branches (see Table 2) were proven to be patent by final control angiograms. Significant or improved intraaneurysmal contrast stasis following FD implantation, quantified according to OKM scale, could be observed in 25 of 34 (73.5%) interventions, of which 6 aneurysms already had a baseline OKM 3 with further improvement therefore not rated. Only 4 aneurysms initially did not show a decrease or prolongation in contrast filling and washout.

**Table 2:** Periprocedural details & measurements

**Aneurysm #**	**Parental Vessel Diameters [mm]**			Mainetance Antiplatelet Protocol		Devices [Diameter x Length, mm]		OKM Stasis		Covered Side-Vessels	Area of Incomplete Vessel Wall Apposition
	Proximal	Distal	Max.			FD Deployed	FD Previously Failed	Baseline	Result		
	Landing Zone										
1	4.5	3.3	4.5	ASA	Ticagrelor (4mo)	4.5 x 15	-	A1	A2	OA	-
2	4.3	2.9	4.3	ASA	Ticagrelor (3mo)	4.5 x 15	-	A1	A1	OA	-
3	3.7	2.8	3.7	ASA	Clopidogrel (3mo)	4.0 x 20	-	A1	A3	OA	-
4	4.0	3.2	4.0	ASA	Ticagrelor (3mo)	4.0 x 15	-	C3	C3	OA	-
5	4.4	3.5	4.4	ASA	Clopidogrel (3mo)	4.5 x 15	-	A2	A3	OA	-
6	3.9	3.1	3.9	ASA	Clopidogrel (3mo)	4.5 x 20	-	A2	?*	-	-
7	3.3	3.2	3.3	ASA	Prausgrel (4mo)	3.5 x 15	-	A2	A2	-	-
8	3.6	2.8	4.3	ASA	Clopidogrel (3mo)	4.5 x 15	-	A2	A3	Pcom + AchA + A1	middle stent portion
9	3.7	2.9	4.1	ASA	Ticagrelor (3mo)	4.0 x 20	-	A1	A3	OA	proximal landing zone (curvature)
10	4.2	2.7	5.1	ASA	Plavix (4mo)	4.5 x 20	D2H 4.5 x 25 (insufficient opening, twist)	A1	A1	OA	middle stent portion
11	3.4	2.4	fusiform	ASA	Ticagrelor (4mo)	4.5 x 25	-	A2	A2	OA + Pcom + AchA + A1	middle stent portion
12	3.7	3.3	4.4	ASA	Clopidogrel (3mo)	5.0 x 25	-	A1	A1	-	-
13	4.1	3.6	4.2	ASA	Ticagrelor (4mo)	4.0 x 15	-	A1	A2	-	proximal landing zone (curvature)
14	3.0	3.0	3.8	ASA	Clopidgrel (4mo)	4.0 x 15	-	A1	A2	OA	-
15	4.0	3.4	4.0	ASA	Ticagrelor (4mo)	4.5 x 15	-	A3	A3	OA	proximal landing zone (curvature)
16	5.3	2.7	fusiform	ASA	Clopdigrel + Dabigatran unitl FU	7.0 x 40	-	A2	A2	P1 right + SCA bilateral (from aneurysm)	-
17	4.6	4.2	4.6	ASA	Clopidgrel (4mo)	4.5 x 15	-	A1	A1	OA	proximal landing zone (curvature)
18	5.6	6.2	fusiform-dysplastic	ASA	Clopidogrel (4mo)	5.0 x 30	D2H 5 x 60 (insufficient opening)	A3	A3	-	proximal landing zone (no curvature)
19	4.4	4.1	4.4	ASA	Clopidgrel (4mo)	4.5 x 25	D2H 4.5 x 25 (insufficient retrieval to sheath)	A3	A3	-	-
20	3.7	2.7	fusiform-dysplastic	ASA	Clopidogrel (3mo)	4.0 x 25	-	A1	A2	A1 + AchA (from aneurysm)	-
21	2.8	3.1	3.5	ASA	Ticagrelor (3mo)	4.0 x 15	-	A1	A2	OA + AchA (from aneurysm neck)	proximal landing zone (curvature)
22	3.8	3.4	3.8	ASA	Clopidogrel (3mo)	4.0 x 15	-	A1	A2	OA	proximal landing zone (curvature)
23	3.2	2.9	3.9	ASA	Ticagrelor (3mo)	4.0 x 20	-	A2	A2	OA	middle stent portion
24	5.2	5.6	fusiform	ASA	Clopdigrel (6mo) + Apixaban (until FU)	6.0 x 30	-	A2	A3	PICA	-
25	3.8	3.5	3.9	ASA	Clopidogrel (3mo)	4.0 x 15	D2H 3.5 x 15mm (too small)	A1	A3	OA	-
26	3.8	2.9	3.8	ASA	Clopidogrel (3mo)	4.0 x 20	-	A1	A2	OA	-
27	3.7	4.3	4.3	ASA	Clopidogrel (3mo)	4.5 x 15	-	A1	A2	OA	proximal landing zone (curvature)
27	3.7	2.9	4.3	ASA	Clopidogrel (3mo)	4.5 x 20	D2H 4.5 x 25 (insufficient opening, twist)	A2	A3	OA (2x)	proximal landing zone (curvature)
28	3.4	3.6	3.6	ASA	Clopidogrel (3mo)	4.0 x 15	-	A1	A2	OA	-
29	5.3	4.1	fusiform	ASA	Ticagrelor (3mo)	5.5 x 30		A3	A3	OA	-
30	4.5	3.1	4.5	ASA	Ticagrelor (6mo)	4.5 x 15		A1	A2	OA	-
31	4.1	3.5	4.6	ASA	Ticagrelor (3mo)	4.5 x 15	D2H 4.5 x 20 (too long)	A1	A2	OA	-
32	3.3	3.4	3.4	ASA	Ticagrelor (3mo)	4.0 x 15		A1	A3	OA	proximal landing zone (curvature)
33 / 34	4.3	3.6	4.3	ASA	Clopidgrel (4mo)	5.0 x 25		A3	A3	OA	proximal landing zone (curvature)

Parent vessel diameters have been measured (based on DSA projection with maximal diameter). Final FD wall adaptation was quantified based on DSA and rotational angiography. Intraaneurysmal stasis was quantified by OKM grading before and after FD deployment. Maintenance antiplatelet protocol included lifelong ASA treatment supplemented by an ADP-receptor antagonist (and an additional anticoagulant in two cases) as illustrated. D2H, Derivo 2 Heal Embolization Device; OKM, O’Kelly-Marotta grading scale; OA, ophthalmic artery; Pcom, posterior communicating artery; AchA, anterior choroidal artery; SCA, superior cerebellar artery; A1, A1 segment of anterior cerebral artery; P1, P1 segment of posterior cerebral artery. * - not possible to quantify based on a control angiogram too short in time

### Periprocedural adverse events

Ischemic complications occurred in 3 of 34 treatments (8.8%), thereby referring to two cases of fusiform posterior circulation aneurysms and to only one out of 31 cases of internal carotid artery aneurysms (3,2%): One patient treated for a paraophthalmic ICA aneurysm developed minor hypoesthesia of the left arm due to microembolic events in the postcentral cortex in the right hemisphere (mRS [modified ranking scale] 0, NIHSS [national institute of health stroke scale] 0). Another two ischemic complications occurred during or immediately after treatment of large fusiform aneurysms (#16/24, see Table 1) in the posterior circulation: One patient treated for a fusiform V4-aneurysm suffered from thalamic microembolic infarctions resulting in transient aphasia (finally discharged with NIHSS 0, mRS 2). Another patient with a fusiform basilar artery aneurysm developed partial in-stent thrombosis with microembolic ischemia (PCA and BA perforator territories) as demonstrated by magnetic resonance imaging (MRI) and digital subtraction angiography (DSA) imaging from the third post-interventional day onwards. The in-stent thrombosis regressed under heparinization, the patient was discharged with persistent internuclear ophthalmoplegia and nystagmus without other symptoms (NIHSS 1, mRS 2).

One patient (aneurysm #27, see Table 3) with postinterventional incomplete wall apposition of the proximal FD was noticed with progressive fish mouthing by control CT performed for headaches on the following day and therefore was treated with a second overlapping, proximally extending D2HED (see Fig. 1-B1/2/3/4). This patient did not show clinical symptoms at any time.

### Imaging follow-up: aneurysm occlusion efficacy & late complications

A first imaging follow-up (CTA or MRI or DSA) after the treatment-associated hospitalization was available for 22 out of 34 aneurysms (64.7%, 77–266 days after intervention, mean 167+/-67 days)^1^, an additional second imaging follow-up was available for another nine aneurysms (26.5%, 111–722 days after intervention, mean 298+/-227 days). A mid-term follow-up (> 5.5 months / >165 days after intervention, as per definition) was available for 16 out of 34 aneurysms (47%), here with a mean follow-up period of 280 +/-160 days. In case of follow-up DSA, aneurysm occlusion was quantified according to OKM grading. In case of follow-up CTA / MRI, aneurysm occlusion was qualitatively assessed as far as comparable to baseline examinations.

**Fig. 2:**
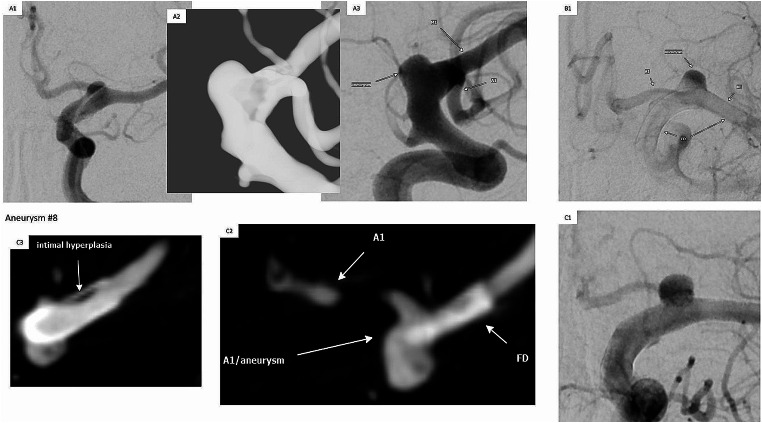
Case Illustration with Intimal Hyperplasia & Missing Aneurysm Occlusion. *(A1-A3)*: Baseline illustration of the terminal ICA aneurysm extending to the vascular outlets of A1/M1 segments. *(B1)*: Control angiogram after FD deployment with contrast media stasis within the aneurysm and slightly delayed perfusion of the A1 segment. *(C1-C3)*: Missing / pending aneurysm occlusion 189 days after FD deployment (C1) supposedly caused by a prominent A1 segment connected to the aneurysm basis (FPCTA C2), furthermore a non-stenosing intimal hyperplasia (FPCTA C3). ICA – internal carotid artery; FD, flow diverter; DSA, digital subtraction angiography; FPCTA, flat panel computed tomography angiography

Based on available follow-up imaging, only 2 aneurysms did not show progressive occlusion or progressive intraaneurysmal stasis: One aneurysm most likely due to very early CTA follow-up 38 days after treatment, performed outside of follow-up routine due to headaches. The other case was a terminal ICA aneurysm with persistent perfusion 189 days after FD implantation supposedly caused by a prominent A1 segment arising from the aneurysm neck (see Fig. 2) itself which presumably promotes trans-/intraaneurysmal blood flow and therefore inhibits or significantly delays aneurysm occlusion or aneurysm regression (further follow-up still pending). All other aneurysms with follow-up showed a decreasing perfusion and/or progressive thrombosis and/or aneurysm size regression based on the available follow-up imaging: 15 totally occluded aneurysms (based on follow-up 111–722 days after intervention) and 5 partially occluded aneurysms (based on follow-up 110–372 days after intervention).

**Fig. 3:**
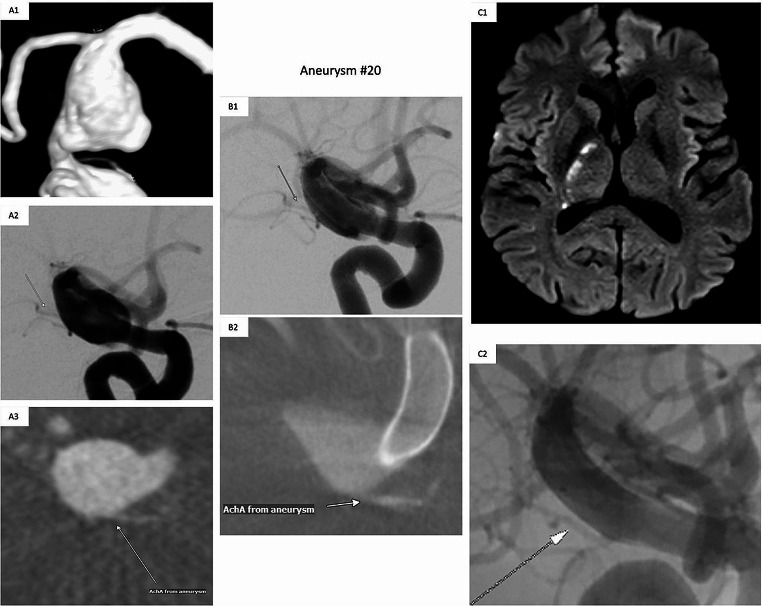
Case Illustration with Ischemic Events in the AchA Territory. *(A1-A3, aneurysm characteristics before FD deployment)*: Baseline imaging with a terminal ICA aneurysm and the AchA (arrows) arising from the aneurysm sac. *(B1/2, DSA and FPCTA images immediately after FD deployment)*: Control angiogram and control FPCTA after flow diverter deployment with the AchA (arrows) proven to be patent. *(C1/C2, 133 days after FD treatment)*: Ischemic areas in the AchA territory (MRI DWI) 133 days after FD treatment and shortly after reduction to sAPT, supposed to be caused by a AchA arising from the progressively thrombosed, initially fusiform-dysplastic aneurysm. Nevertheless, the AchA was proven to be still patent in the subsequent follow-up DSA (C2). AchA, anterior choroidal artery; ICA – internal carotid artery; FD, flow diverter; MRI, magnetic resonance imaging; DWI, diffusion weighted imaging; DSA, digital subtraction angiography; FPCTA, flat panel computed tomography; APT, antiplatelet therapy

Regarding complications based on follow-up imaging, there was one patient (aneurysm #20) with acute AchA ischemia 133 days after FD deployment for a fusiform-dysplastic ICA aneurysm with the AchA arising from the aneurysm itself. This ischemic event occurred ten days after Clopidogrel was stopped (see Fig. 3). Despite the AchA ischemia, the AchA itself was proven to be open with an aneurysm totally occluded based on MRI and angiography. Therefore, a small thrombembolus was suspected to have traveled downstream into the distal AchA and then resolved. Furthermore, two FDs showed a mild intimal hyperplasia (non-stenotic < 25%) based on DSA 189 / 257 days after FD implantation. No other follow-up complications were observed. Interestingly, one out of 11 FDs with initially incomplete apposition of the proximal FD within a parental vessel curvature was followed-up by DSA and showed a remodeling / retraction with completed vessel wall apposition (see Fig. 1-A1/2).

**Table 3:**
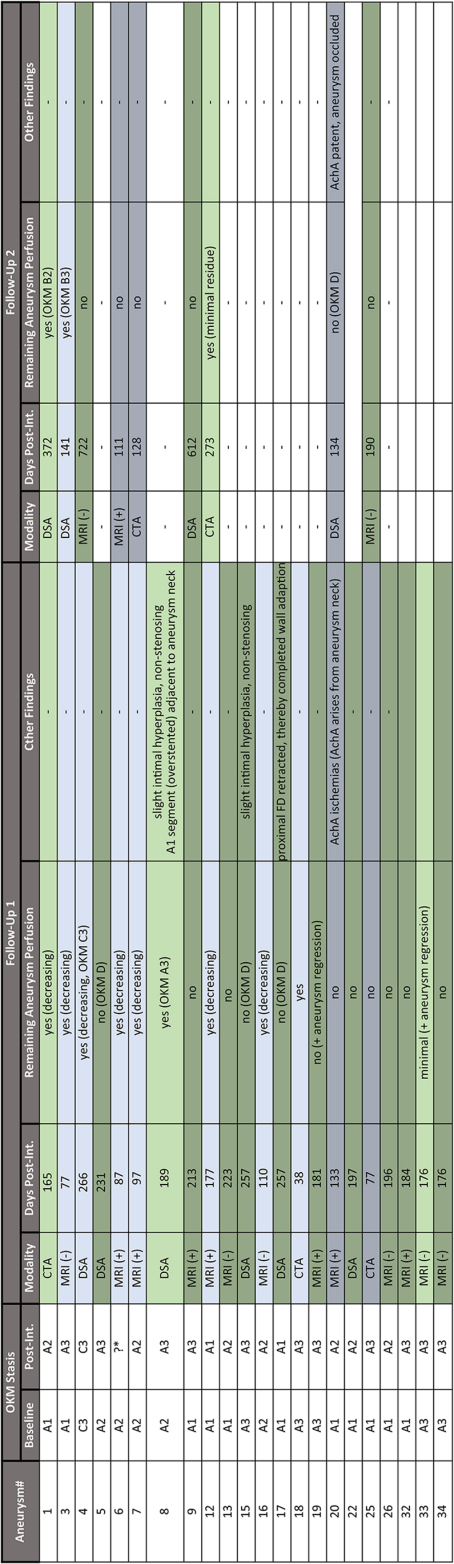
Follow-up assessment

Available follow-up imaging is differentially considered as short-term (<165 days, blueish highlighted) and mid-term (at least 165 days after intervention, greenish highlighted) and aneurysms with proven complete occlusion are highlighted by a background line pattern. In the case of follow-up MRI/MRA or CTA, the remaining perfusion of the aneurysm was qualitatively assessed. If follow-up DSA data was available, the remaining aneurysm perfusion was quantified using OKM grading. Other follow-up findings related to the FD, treated aneurysm and downstream vascular territory have been recorded. FD, flow diverter; OKM, O’Kelly-Marotta grading scale; AchA, anterior choroidal artery; CTA, computed tomography angiography; MRI (-), magnetic resonance imaging without contrast media enhancement; MRI (+), magnetic resonance imaging with contrast media enhancement. DSA, digital subtraction angiography. * - not possible to quantify based on a control angiogram too short in time

## Discussion

Our preliminary pilot study results demonstrate periprocedural safety as well as follow-up efficacy of the antithrombogenic coated D2HED flow diverter for the treatment of intracranial aneurysms. There is only one similar case series published so far, nevertheless with a significantly lower number of only 9 patients included and especially only 2 patients with a follow-up period of at least six months [14]. We are sharing the largest and, so far, the only case series including a relevant number of mid-term follow-ups.

Regarding periprocedural feasibility: All cases were treated by finally successful FD deployment but at a rate of 10% with a not completely satisfactory opening behavior (*n* = 3) or failed retrieval before intended repositioning (*n* = 1) that we attribute to the choice of long FD sizes in or near the carotid siphon. Objective comparisons to other studies, which may not similarly report such cases, are also complicated by the fact that in cases of unsatisfactory initial opening behavior, the neurointerventionalist’s decision to retrieve or to release and manipulate the device remains highly individualized. Nevertheless, unsatisfactory FD opening behavior is a well-known phenomenon and is also described for other flow diverter devices, such as exemplarily for the *Silk Vista* with 9% [17] or for the p64 HPC with 6% (when summing up torsion and incomplete opening cases according to Ernst et al. [18]).29% of D2HED required some form of mechanical manipulation to improve wall apposition – an information often not quantified by comparative studies (e.g., Pinana et al. [19]). Ongoing incomplete parental vessel wall apposition was predominantly observed at the proximal FD landing zone and in parent vessel curvatures. None of these suboptimal wall appositions had a clinical impact. Follow-up DSA available for one of these patients with a proximal FD non-apposition showed spontaneous FD shortening / remodeling with resulting complete wall adaptation in the meantime, another asymptomatic patient with presumably progressive fish mouthing in the early postinterventional course received an early retreatment by proximal FD extension using a second D2HED. Significant flow reduction and/or immediately reduced intraaneurysmal blood flow was seen with 25/34 (73.5%) D2HED deployments resulting in quantifiable flow diversion seen as intraaneurysmal contrast stasis. This is comparable, e.g., with 82.1% reported contrast retention immediately after deployment of the non-coated Derivo Embolization Device as demonstrated by Pinana et al. based on a large multicenter study [19]. Furthermore, all treating interventionalists subjectively found the D2HED to demonstrate good fluoroscopic visibility.

Regarding aneurysm occlusion efficacy based on follow-up imaging: One patient did not show aneurysm occlusion even 189 days post treatment, presumably caused by a promiment A1 segment arising from the fusiform-dysplastic aneurysm leading to an aneurysm perfusion maintenance. Excluding this patient, all other aneurysms followed-up at least 77 days after treatment showed a progressive occlusion. A partial occlusion with a partially maintained aneurysm perfusion at mid-term follow > 5.5 months / > 165 days after treatment was demonstrated for three aneurysms, all other aneurysms showed complete occlusion at latest at mid-term follow up, resulting in a mid-term occlusion rate of 80%. However, these data should be interpreted with caution given the very small sample size of follow-up cases and due to the underlying heterogeneous follow-up examinations. Especially partially externally conducted follow-up MRIs (of which some – contrary to our recommendations – have been acquired only with TOF-MRA without contrast-enhanced sequences) may possibly underestimate a minor aneurysm perfusion. However, disregarding the mentioned limitations, a mid-term complete occlusion range (COR) at > 5.5 months roughly estimated at 80% would be (as far as comparable at low case numbers and differing follow-up periods) in similar ranges compared with the CORs of other established FDs [20] – e.g. the *Pipeline Embolization Device (PEP)* with a 6-month-COR of 93.6% [21], the *Pipeline Flex Embolization Device* with a 1-year-COR of 76.8% [22], the *Surpass Evolve* with a 4-months-COR of 57% [23], the *Fred* with a 1-year-COR of 91.3/73.3% [24, 25], the *Silk* with a 1-year-COR of 93.9% [26], the p64 with a 6-months-COR of 66.6%/82% [27, 28] or the *Derivo* with a 6-months-COR of 80.7% and a 1-year-COR of 89.2% [29].

Regarding the efficacy of the D2HED fibrin-/heparin-based surface nano-coating, especially the two cases of mild, non-stenotic intimal hyperplasia and one case of early in-stent-thrombosis need to be discussed and compared to conventional, bare metal FDs. Based on the literature, neointimal hyperplasia was shown to be associated with FD incomplete wall apposition, stent design, surface modifications and cardiovascular risk factors [30–32]. Based on an animal model, the neointimal thickness was shown to not significantly differ between the conventional *Derivo Embolization Device* and the D2HED [33]. Considering the localization of intimal hyperplasia in our study, an initial FD wall dehiscence (incomplete wall apposition) was not observed in either of the two cases. Despite ultimately unexplained reasons for the observed intimal hyperplasia, the incidence in two of our cases (corresponding to 25% in relation to all cases with follow-up DSA available) appears to be relatively low in the literature context, e.g. compared with 68% / 38% at early follow-up (< 12 months) after implantation of *Silk* / *PEP* FDs [32]. For the occurrence of one early in-stent thrombosis despite good dual APT response proven by *multiplate testing*, no periinterventional cause could ultimately identified. An obvious and well-known explanation remains the generally increased thrombogenicity of FDs as compared to conventional stents due to higher metal mesh density.

Regarding complications of clinical impact: We observed three clinically relevant complications in 34 FD deployments (8.8%), differentially distributed across complication rates of 3,2% (1/31) regarding ICA aneurysms (one minor ischemic event) and 66.6% (2/3) regarding aneurysms in the posterior circulation (one minor ischemic event and another case with in-stent thrombosis as well as minor ischemic event, both cases referring to fusiform aneurysms in the posterior circulation). This appears to be an acceptably low complication rate compared with e.g. an overall FD-related complication rate of 17.0% (95%CI 13.6–20.5%) as demonstrated by a meta-analysis including 3125 patients predominantly treated with *PEP* or *Silk* flow diverter devices [34].

Limitations of this study mainly refer to its retrospective single-center design with a relatively small number of enrolled patients, especially regarding the limited number of patient-individual follow-up controls including different modalities and heterogeneous MRI protocols with partially lacking contrast-enhanced sequences. In this context, it must also be mentioned that while MRI follow-up can at least qualitatively estimate remaining aneurysm perfusion to some extent, it cannot exclude other, potentially clinically silent complications (such as fish mouthing or intimal hyperplasia). The consecutive patient inclusion without exclusion criteria promotes clinical representativeness, although it must be acknowledged that the resulting study cohort may also have institution-specific characteristics: For instance, ruptured aneurysms as well as other common aneurysm locations (such as MCA, Acom or basilar tip) are not well represented for clinical reasons, e.g., to avoid dual antiplatelet therapy in ruptured cases or due to the preference for other strategies than flow diverters in other aneurysm locations. Nevertheless, other larger multicenter studies using a similar strategy of patient inclusion finally presented comparable cohort characteristics, e.g., Pinana et al. [19] with 250 aneurysms of which 87% with ICA localization, only 1% ruptured cases, and less than 5% of aneurysms with sizes exceeding 20mn. Also, the cohort heterogeneity including fusiform vertebrobasilar aneurysms must be taken into account, for instance, when considering the overall complication rate, to which the low proportion of fusiform aneurysms in the posterior circulation contributes disproportionately. Another aspect limiting the study is the use of D2HED as the sole inclusion criterion while giving treating neuroradiologists the freedom to choose between different flow diverters, which may introduce potential study bias. Furthermore, investigating the effect of the antithrombogenic surface coating of the *D2HED* with a possible impact on thrombogenic complications and necessary APT should be the focus of further studies.

## Conclusion

Our preliminary pilot results based on a series of 34 D2HED deploying interventions demonstrate the periprocedural feasibility of the *Derivo 2 Heal Embolization Device* as compared to performance data of other flow diverters based on literature research. A conclusive statement regarding aneurysm occlusion efficacy cannot be drawn from our dataset comprising only 16 mid-term follow-up cases, yet these suggest a competitive mid-term occlusion rate estimated at 80%. Further studies of higher case numbers are warranted, also focusing on longer-term follow-up and the clinical impact of the surface coating on thrombogenic complications and necessary antiplatelet therapy.

## Footnotes

1 Nine aneurysms were treated shortly before the completion of the study, resulting in pending follow-up imaging. Three patients (among them the ruptured aneurysm of a patient also suffering from cancer) did not attend the follow-up for reasons that could not be further clarified.
